# Si-rich Silicon Nitride for Nonlinear Signal Processing Applications

**DOI:** 10.1038/s41598-017-00062-6

**Published:** 2017-02-02

**Authors:** Cosimo Lacava, Stevan Stankovic, Ali Z. Khokhar, T. Dominguez Bucio, F. Y. Gardes, Graham T. Reed, David J. Richardson, Periklis Petropoulos

**Affiliations:** Optoelectronics Research Centre, University of Southampton, SO17 1BJ Southampton, UK

## Abstract

Nonlinear silicon photonic devices have attracted considerable attention thanks to their ability to show large third-order nonlinear effects at moderate power levels allowing for all-optical signal processing functionalities in miniaturized components. Although significant efforts have been made and many nonlinear optical functions have already been demonstrated in this platform, the performance of nonlinear silicon photonic devices remains fundamentally limited at the telecom wavelength region due to the two photon absorption (TPA) and related effects. In this work, we propose an alternative CMOS-compatible platform, based on silicon-rich silicon nitride that can overcome this limitation. By carefully selecting the material deposition parameters, we show that both of the device linear and nonlinear properties can be tuned in order to exhibit the desired behaviour at the selected wavelength region. A rigorous and systematic fabrication and characterization campaign of different material compositions is presented, enabling us to demonstrate TPA-free CMOS-compatible waveguides with low linear loss (~1.5 dB/cm) and enhanced Kerr nonlinear response (Re{*γ*} = 16 Wm^−1^). Thanks to these properties, our nonlinear waveguides are able to produce a π nonlinear phase shift, paving the way for the development of practical devices for future optical communication applications.

## Introduction

Over the last decade, Silicon Photonics has established itself as a mature technology for the fabrication of low-cost, scalable integrated optical components^1^. Silicon-On-Insulator (SOI) has been widely accepted as the ideal fabrication platform for silicon photonics components, allowing the implementation of high-index contrast waveguides using CMOS-compatible processes. A wide range of highly performing SOI-devices aimed at applications for next-generation optical networking have already been demonstrated, such as ultra-low loss waveguides and optical filters^1^, high-speed optical transceivers^2^, as well as components for all-optical signal processing^3,4^. Nonlinear optical processing applications have also been demonstrated, including all-optical wavelength converters^5,6^, signal regeneration^7^, parametric gain^8^ as well as high-speed switching^3^. These applications benefit both from the high nonlinear refractive index of silicon and the tight field guidance achieved within SOI waveguides. However, they suffer at a fundamental level from the very large two-photon absorption (TPA) coefficient of silicon at telecommunication wavelengths^4,9–12^. As a result, TPA-related effects become significant in typical strip waveguides in silicon, even at power levels of a few mWs, thereby preventing the widespread adoption of silicon as a nonlinear medium at these wavelengths (unless some active means for suppressing TPA is adopted^5^).

A number of works have emerged in recent years that explore alternative materials, suitable for the implementation of nonlinear integrated optical devices. These materials include amorphous silicon that possesses enhanced nonlinear performance with respect to its crystalline counterpart exhibiting an increased Kerr coefficient and a reduced TPA effect at telecoms wavelengths^13–17^ and Hydex ®^18^ which exhibits exceptional low linear loss at the 1550-nm wavelength region (α < 0.06 dB/cm) and high Kerr nonlinearity^19^, showing no signs of TPA even at relatively high power levels^20^. *AlGaAs* ^21–25^, *Ta*_*2*_ *O*_*5*_^26,27^ and *SiGe* ^28,29^ have also been studied recently and proposed as CMOS-compatible alternative platforms for the development of integrated nonlinear components. Compatibility with existing SOI technologies is still a primary consideration in these studies. Among the various alternatives, silicon nitride Si_3_N_4_ (where Si_3_N_4_ replaces silicon in the core of the waveguide) has recently been investigated by several research groups^19,30–33^, showing both remarkable linear and nonlinear performance. Silicon nitride also exhibits low absorption in the visible wavelength range (down to 500 nm), adding functionalities that are not possible to achieve with SOI, such as integration with light sources emitting in the visible range, spectroscopic functions and signal up-conversion from the telecom wavelength range^30^. On the other hand, as a material platform, silicon nitride also suffers from certain limitations, such as a reduced waveguide core/ cladding index contrast (0.5, as opposed to 2 for SOI) that may require the use of Watt-level optical powers in order to give rise to high enough nonlinear phase shifts for nonlinear optical applications, as well as fabrication challenges when relatively thick wafer layers are required (thicker than 400 nm)^30,34,35^. In order to enhance the nonlinear response of the material, and thus reduce the optical power required to achieve the desired nonlinear phase-shift, modified compositions of silicon nitride have been recently proposed^36–38^, leading to silicon-rich silicon nitride, which exhibits enhanced Kerr response with respect to the standard, stoichiometric Si_3_N_4_ material composition. However, very high propagation losses (10 dB/cm) were observed in refs 36,37, while the lower-loss device in ref. 38 showed only a modest Kerr response. In this work, we systematically study the optical properties of different compositions of Si_x_N_y_ employing exclusively low temperature (<350 °C) Back End of Line (BEOL) CMOS compatible processes. The proposed engineered platform provides a high index contrast, low-stress material, facilitating TPA-free operation that can be integrated with the existing SOI technology to allow for the implementation of crucial functionalities.

## Fabrication and Results

### Sample fabrication and material characterization

**Figure 1:**
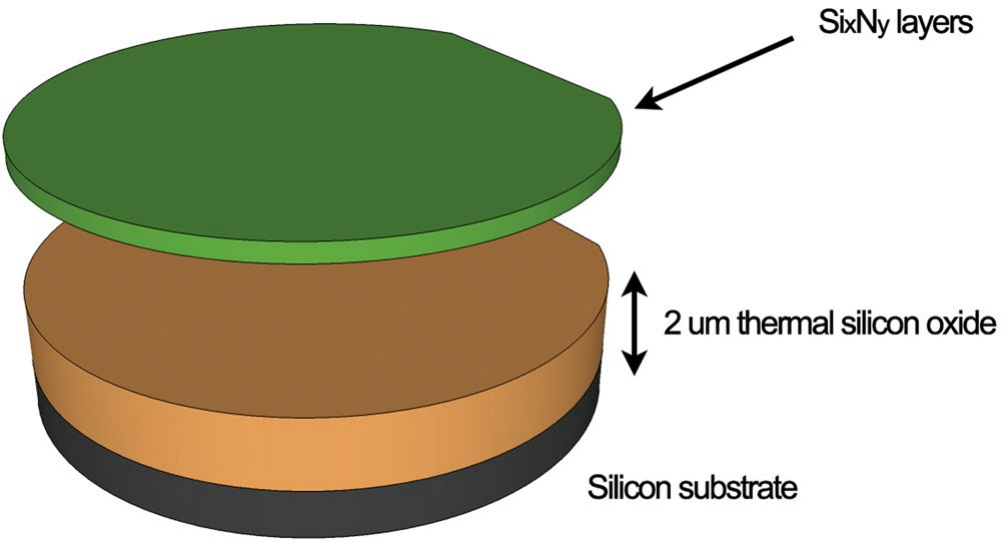
Schematic representation of the wafer composition.

**Figure 2:**
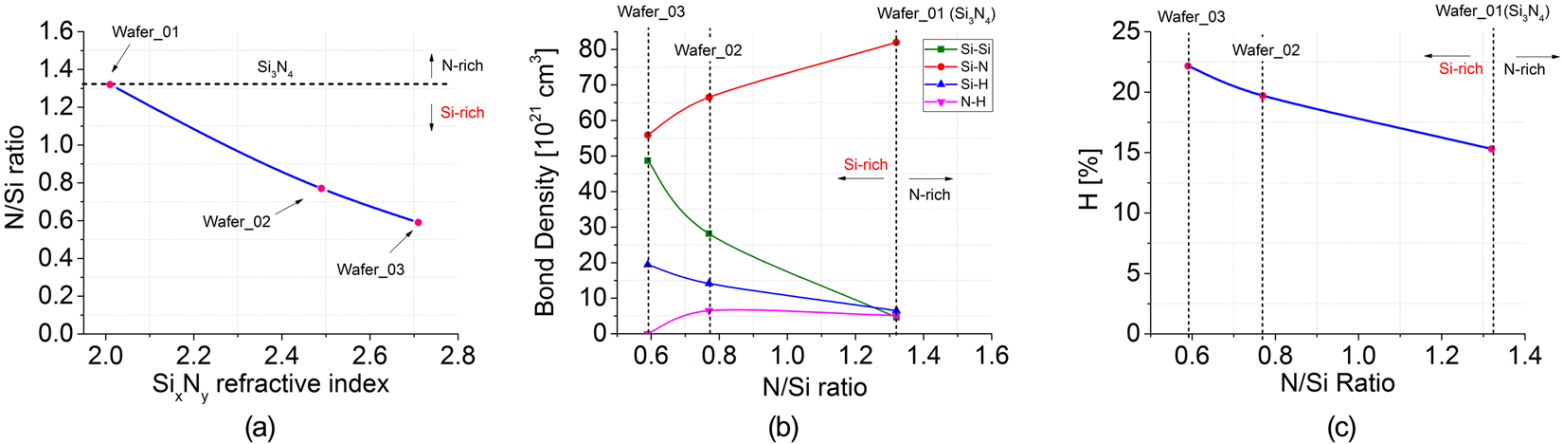
(**a**) N/Si ratio as a function of the Si_x_N_y_ layer refractive index; (**b**) Bond concentrations for each Si_x_N_y_ layer configuration; (**c**) Hydrogen content (in atomic %) for each Si_x_N_y_ layer configuration.

In order to assess the linear and nonlinear optical properties of silicon nitride waveguides, a set of three Si_x_N_y_ materials with different stoichiometric ratios were developed and deposited on a thermally-grown SiO_2_ layer (2 μm thickness) on a 150-mm diameter Si substrate wafer using Plasma Enhanced Chemical Vapour Deposition (PECVD). The three PECVD processes were optimized in order to provide a repeatable deposition thickness, refractive index and uniformity levels across the wafers. A standard SOI wafer was also used to compare the achieved results to the state of the art, SOI technology. A schematic of the layer composition of each wafer is shown in Fig. 1. The material properties and compositional structures of each Si_x_N_y_ were assessed using different characterization techniques: ellipsometry (carried out by means of a M2000DI ellipsometer) was employed to determine the thickness uniformity and refractive index of the layers at 632.8 nm, 1310 nm and 1550 nm and Fourier Transform Infrared Spectroscopy FTIR spectroscopy was used to determine the compositional structure of each fabricated layer, including their bond concentration, atom concentration and hydrogen content. The results collected by ellipsometry (at a wavelength of 1550 nm) are outlined in Table 1. By using these data and the following equation, it is possible to estimate the N/Si ratio within each wafer^39,40^:

1 $$\frac{N}{Si}=(\frac{4}{3})(\frac{{n}_{Si}-n}{n-2{n}_{Si}+{n}_{Si}})$$

where *n* is the measured layer refractive index and *n*_*Si*_ is the refractive index of silicon. Results are presented in Fig. 2(a) showing an increased silicon content in the Wafer_02 and Wafer_03 compositions with respect to the stoichiometric Si_3_N_4_. Knowledge of the bond compositions for each different Si_x_N_y_ layer is useful in order to understand the optical behaviour of the fabricated material, since they strongly influence the material quality, and subsequently the optical material loss. High concentration of Si-H and Si-Si bonds have been shown to be responsible for the formation of undesired grains, pores and columnar microstructures that can cause additional scattering and absorption losses^41,42^. The bond compositions were extracted using the method described by Tin and Smith^41^ and the following proportionality factors: *K*(*N-H*) = 8.2 × 10^16^ cm^−1^, *K*(*Si-H*) = 5.9 × 10^16^ cm^−1^ and *K*(*Si-N*) = 2.4 × 10^16^ cm^−1^ (as provided by Landford and Rand^43^ and Bustarret *et al*.^39^) while the hydrogen, nitrogen and silicon content (atom concentrations [H], [N] and [Si]) were extracted using the following equations^40,41^:

2 $$[{\rm{H}}]=[{\rm{N}}\mbox{-}{\rm{H}}]+[{\rm{S}}{\rm{i}}\mbox{-}{\rm{H}}]$$

3 $$[{\rm{N}}]=([{\rm{N}}\mbox{-}{\rm{H}}]+[{\rm{S}}{\rm{i}}\mbox{-}{\rm{N}}])/3$$

4 $$[{\rm{Si}}]=[{\rm{N}}]/x$$

5 $$[{\rm{S}}{\rm{i}}\mbox{-}{\rm{S}}{\rm{i}}]={\rm{2}}[{\rm{Si}}]-\,\frac{1}{2}([{\rm{S}}{\rm{i}}\mbox{-}{\rm{N}}]+[{\rm{S}}{\rm{i}}\mbox{-}{\rm{H}}])$$

where [N-H], [Si-H] and [Si-N] represent the N-H, Si-H and Si-N bond concentrations, respectively. Results are reported in Fig. 2(b) and the hydrogen content (in atomic %) is shown in Fig. 2(c). Si-Si bonds significantly increase when the layer is rich in silicon (Fig. 2(b)), while reach their lowest level when the material composition is close to the stoichiometric Si_3_N_4._ This is due to the fact that back bonded Si atoms are dominant when N/Si is decreased, while they tend to decrease in favour of N bonds (Si-N or N-H) when N/Si approaches the value of 1.33 (stoichiometric Si_3_N_4_)^42^.

**Table 1:** Wafer description and specification.

Wafer ID	Si_x_N_y_ layer refractive index (at 1550 nm)	Si_x_N_y_ layer thickness (measured at the centre of the wafer)
Wafer 01	2.01	300.5 ± 0.3 nm
Wafer 02	2.49	297.2 ± 0.2 nm
Wafer 03	2.71	308.1 ± 0.4 nm

A number of strip waveguides were patterned on each of the three wafers. The widths of the waveguides varied from 500 nm to 1500 nm (with a 100 nm step) and their lengths from 0.1 cm to 1.1 cm (with a 0.1 cm step). Inductively Coupled Plasma (ICP) etching was used to form waveguide patterns, by performing a single full-etch step down to the underlying SiO_2_ layer, thus forming the desired strip waveguide structures. In order to access each photonic structure, both grating couplers and butt coupling tapers were fabricated. Photonic structures incorporating grating couplers were employed in experiments involving continuous-wave (CW) beams, whereas structures with wide- bandwidth spot size converters for butt-coupling were utilized for pulsed-light experiments, thus avoiding any spectral shaping on the input pulse trains. Every waveguide was patterned 100 times across each wafer and all waveguides were tested, thus enabling statistically consistent optical characterization measurements.

### Linear optical properties

**Figure 3:**
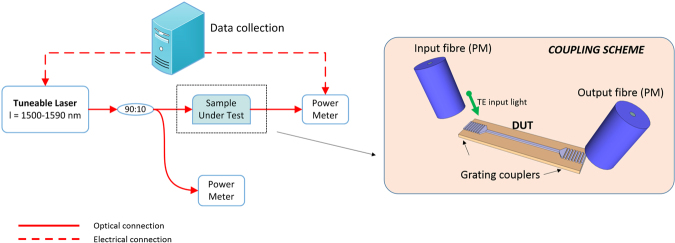
Propagation loss experimental scheme. PM: Polarization Maintaining; DUT: Device Under Test.

The waveguide propagation loss is an important factor in determining the nonlinear efficiency of a photonic device. This is because the effective device length *L*_*eff*_ that contributes to its nonlinear response is a function of its propagation loss *α*. In the extreme of a high-loss waveguide, the effective length approaches 1/*α*, i.e. it is the propagation loss rather than the physical device length L that determines *L*_*eff*_^44^. In high index contrast waveguides (e.g. Δn > 0.5) it is typically influenced by three main factors: a) the quality of the optical material, b) the material absorption and c) surface roughness on the sidewalls and its interaction with the optical mode. Experimental measurements of the propagation loss were carried out by employing the cut-back technique, using the experimental set-up shown in Fig. 3. A tuneable CW laser source was used to carry out measurements across the 1500 nm–1590 nm wavelength region. Light coupling, both in and out of the waveguides, was achieved by grating couplers whose bandwidth was centred at λ = 1550 nm (with a 3-dB bandwidth of 40 nm). The polarization of the input beam was set to the TE state and was maintained through a fully polarization maintaining set-up.

**Figure 4:**
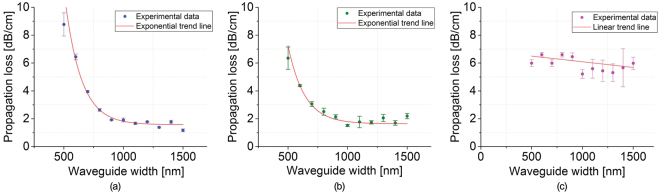
Waveguide propagation losses for (**a**) Wafer 01, (**b**) Wafer 02 and (**c**) Wafer 03.

Loss measurements were carried out on each sample, for each silicon nitride composition, thus assessing the effect of different silicon contents, within the Si_x_N_y_ material. The results of these measurements are presented in Fig. 4, where each point on the graphs represents the average propagation loss value (over 100 samples) measured for each waveguide width configuration. Error bars are also shown, which take into account systematic errors (due to the measurement procedure) as well as loss uniformity across the whole wafer (please refer to the Methods section for more information).

Both Wafers 01 and 02, exhibiting the lowest refractive indices, show the typical exponential dependence of loss versus waveguide width, with narrower waveguides experiencing higher losses due to a greater interaction between the optical mode and the sidewalls. The higher refractive index contrast Wafer 03 does not show this behaviour, and we believe this to be because the optical mode is well confined within the waveguide core even at waveguide widths as small as 500 nm.

The results highlight that PECVD Si-rich silicon nitride waveguides with propagation losses approaching 1.5 dB/cm were realized. Increased propagation losses were measured for waveguides grown on Wafer 03 (6 dB/cm, see Fig. 4(c)) showing that an excessive Si content in the material composition leads to reduced material quality. Since loss results presented in Fig. 4(c) do not show a significant dependence on the waveguide width, we believe that the majority of the optical losses observed are material losses. PECVD-grown Si_x_N_y_ layer losses, at λ = 1550 nm, are typically influenced by three main factors: (a) the hydrogen content that significantly increases the number of both N-H bonds, which cause an absorption peak at a wavenumber of 3350 cm^−1^ (first overtone corresponds to λ ~ 1500 nm with low energy tails to 1545–1560 nm) and Si-H bonds, with an absorption peaking at a wavenumber of 2300 cm^−1^ (second overtone corresponding to λ ~ 1490 nm with low energy tails extending to the C-band wavelength region)^45–47^; (b) Si-Si and Si-H bond concentrations that have been identified as a possible cause for the generation of additional silicon dangling bonds which are responsible for additional absorption; (c) material microstructure that may show grains, pores, defects, cluster and voids generated by high concentrations of [H]^48^, [Si-Si] and [Si-H] bonds^42^. Wafer_03 material composition showed the highest concentration of both Si-Si and Si-H bonds (Fig. 2) and an increased concentration of hydrogen with respect to Wafer_02 and Wafer_01. We believe that the high concentration of Si-Si and Si-H bonds are contributing to the formation of unwanted silicon dangling bonds and the formation of undesired defects within the material (such as pores clusters and grains that can act as scattering centres)^42,49^ that are also favoured by the highest presence of both silicon and hydrogen (Fig. 2). All of these factors contribute to compromise the optical quality of Wafer_03 composition, leading to a relatively high loss material.

### Nonlinear optical properties

A systematic characterization of the nonlinear optical properties of the waveguides, fabricated in the three different wafer compositions is presented in this section. As discussed previously, one of the main drawback of silicon-based nonlinear devices is the strong presence of TPA-related effects that restrict the use of such devices to relatively low power levels, practically reducing the device nonlinear efficiency. Here we show that silicon nitride layers can be properly engineered to show a high Kerr nonlinearity response, with no TPA-related effects, allowing waveguides to be operated at Watt-power levels.

The waveguide nonlinear response can be generally described by the nonlinear parameter γ which is defined by the following expression^50^

6 $$\gamma (\omega )=\frac{3\omega }{4{\varepsilon }_{0}{c}^{2}{A}_{eff}{{n}_{eff}}^{2}}{\chi }^{(3)}$$

where *ε*_0_ is the vacuum permittivity, *χ* ^(3)^ the third order susceptibility, *n*_*eff*_ is the modal effective refractive index at frequency *ω* and *A*_*eff*_ is the mode area of the considered waveguide. It is important to note that, in general, *χ* ^(3)^ is a complex number and, therefore, *γ* also has a real and an imaginary part and can be written as:

7 $$\gamma (\omega )=\mathop{\underbrace{\omega {n}_{2}/(c{A}_{eff})}}\limits_{{\rm{Re}}\{\gamma \}({\rm{Kerr}}\,{\rm{nonlinearity}})}+\mathop{\underbrace{i{\beta }_{TPA}/(2{A}_{eff})}}\limits_{{\rm{Im}}\{\gamma \}({\rm{nonlinear}}\,{\rm{loss}})}$$

where *n*_2_ represents the nonlinear refractive index and *β*_*TPA*_ is the TPA coefficient defining the strength of the nonlinear loss of the waveguiding device.

**Figure 5:**
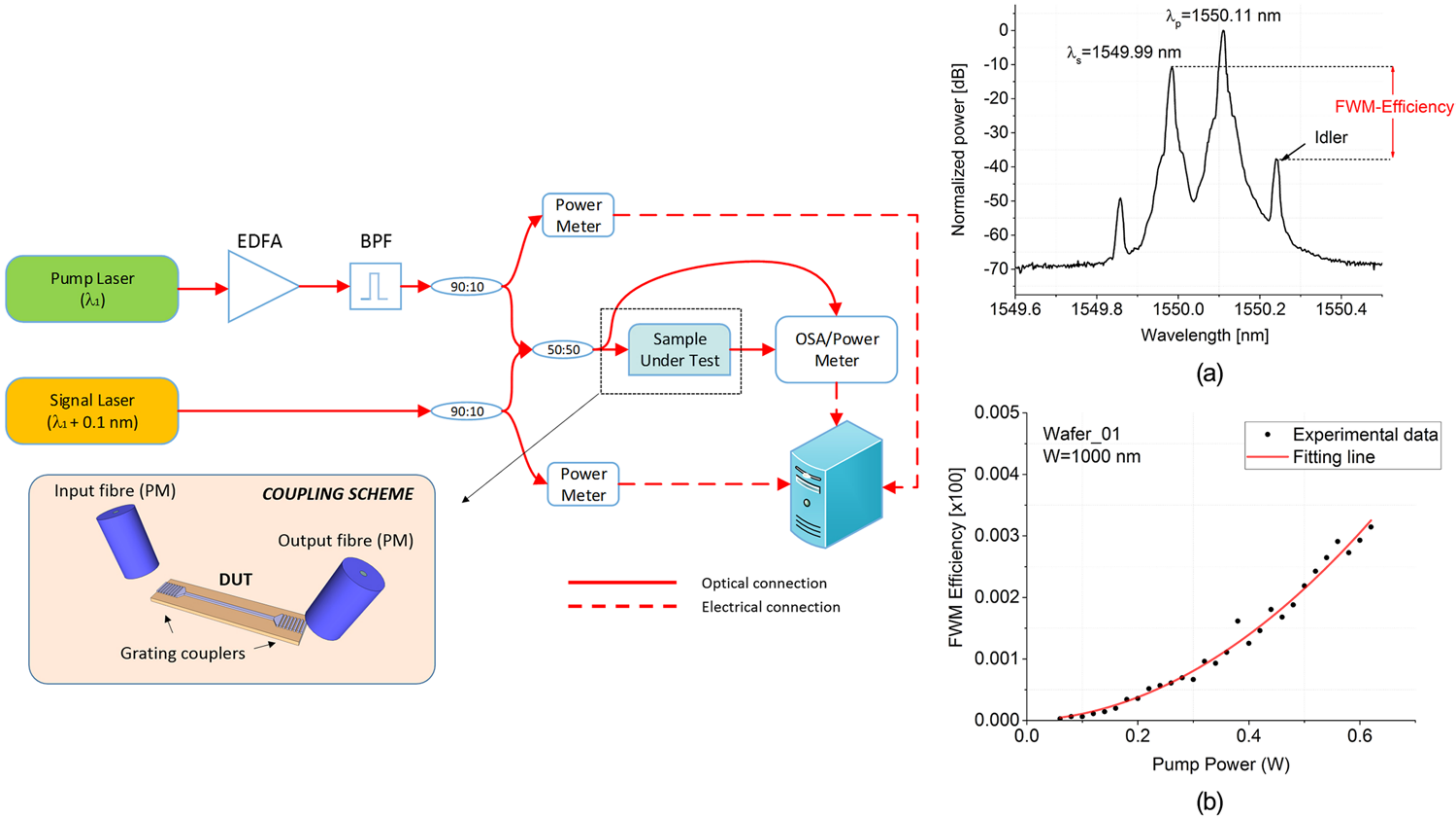
CW-FWM experimental set-up; BPF: Band Pass Filter. (**a**): example of CW-FWM spectrum recorded at the output of the waveguide (Wafer_02, W = 1000 nm, P_p_ = 320 mW); (**b**): example of FWM-efficiency curve versus optical power used to calculate the Re{γ} coefficients (Wafer_01, W = 1000 nm).

Re{γ} can be assessed by adopting a CW-FWM-based scheme^22^. As presented in the experimental set-up of Fig. 5, a pump laser (set at a wavelength of λ_p_ = 1550.11 nm, power ranging from 20 mW to 2 W before the waveguide input grating coupler) was amplified by a polarization maintaining Erbium-doped fibre amplifier (PM-EDFA) and was sent to an optical band pass filter to remove the amplified spontaneous emission (ASE), originated by the EDFA. The band pass filter (BPF) showed a 3 dB bandwidth of 0.078 nm and a 10 dB bandwidth of 0.145 nm. A fibre coupler (50:50) was used to combine the pump signal with a weaker CW light beam originated from a tuneable external cavity laser (ECL) and set at a wavelength of λ_s_ = 1549.99 nm. The optical power of the signal beam (λ_s_) was always kept at least 10 dB lower than the pump signal (λ_p_) power level. The two waves were coupled to the waveguide under test by means of grating coupler devices, as depicted in Fig. 5 (bottom panel). Light polarization was set to TE for both pump and signal beams, and maintained constant along the experimental scheme by utilizing PM fibre components. FWM in the waveguide generated an idler at a new frequency and all waves were coupled back to a PM fibre through the output grating coupler. Spectra were recorded for different pump power levels, allowing the Re{*γ*} coefficients of the fabricated devices to be extracted, according to the following formula:

8 $$\mathrm{Re}\{\gamma \}=\frac{\sqrt{{P}_{i}(L)/{P}_{s}(L)}}{\eta {P}_{p}(0){L}_{eff}}$$

where *P*_*i*_(*L*) and *P*_*s*_(*L*) are the idler and signal power levels, measured at the output of the waveguide under test, respectively; *P*_*p*_(0) represents the pump power measured at the input of the waveguide, *L*_*eff*_ is the nonlinear effective length^44^ and *η* accounts for the phase-mismatch induced by chromatic dispersion. By placing the signal and pump beams relatively close to each other in wavelength (*Δλ* < 0.15 nm) the effect of dispersion can be neglected, thus allowing to consider *η* = 1. The experimental scheme was calibrated and validated by measuring a set of standard silicon waveguides fabricated in our cleanroom starting from a commercial SOI wafer (channel waveguides, width = 500 nm, height = 220 nm). Figure 5(a) shows a typical recorded spectrum (Wafer_02, W = 1000 nm, *P*_*p*_ = 320 mW) while an example of a FWM efficiency-vs-pump power curve (Wafer_01, W = 1000 nm) is shown Fig. 5(b), revealing the typical quadratic behaviour of such effects^44^.

**Figure 6:**
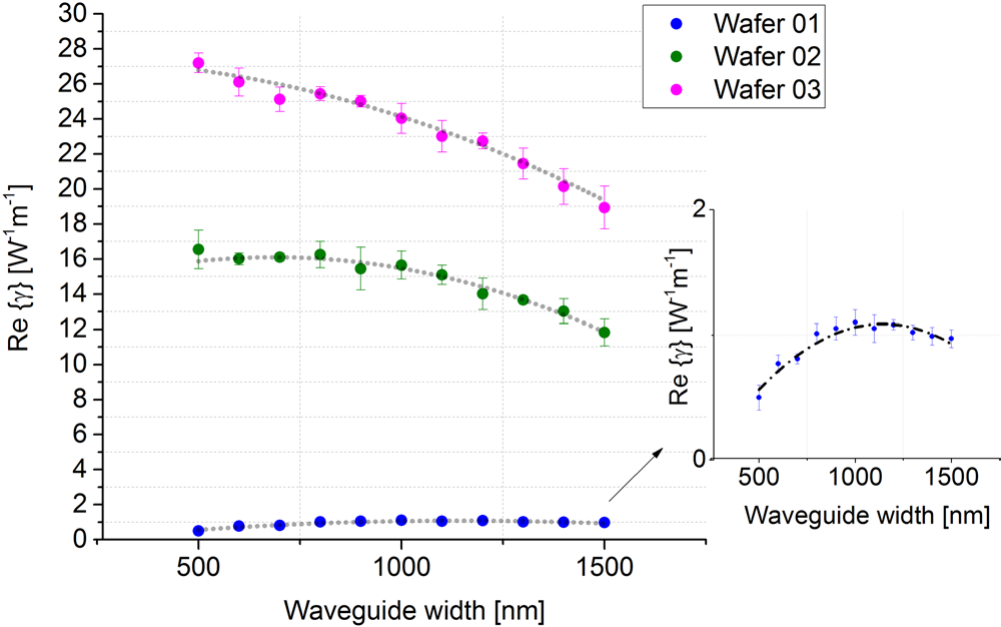
Main panel: Re{γ} measured for each waveguide configuration, on each silicon nitride composition. Inset: zoom of Wafer 01 Re{γ} measurements, to clearly show the extent of the error bars.

The graph in Fig. 6 compares the values of Re{*γ*} for the waveguides in the three different wafers. The error bars were calculated through measurements on the 100 different waveguides of the same dimension that were written on each wafer.

**Figure 7:**
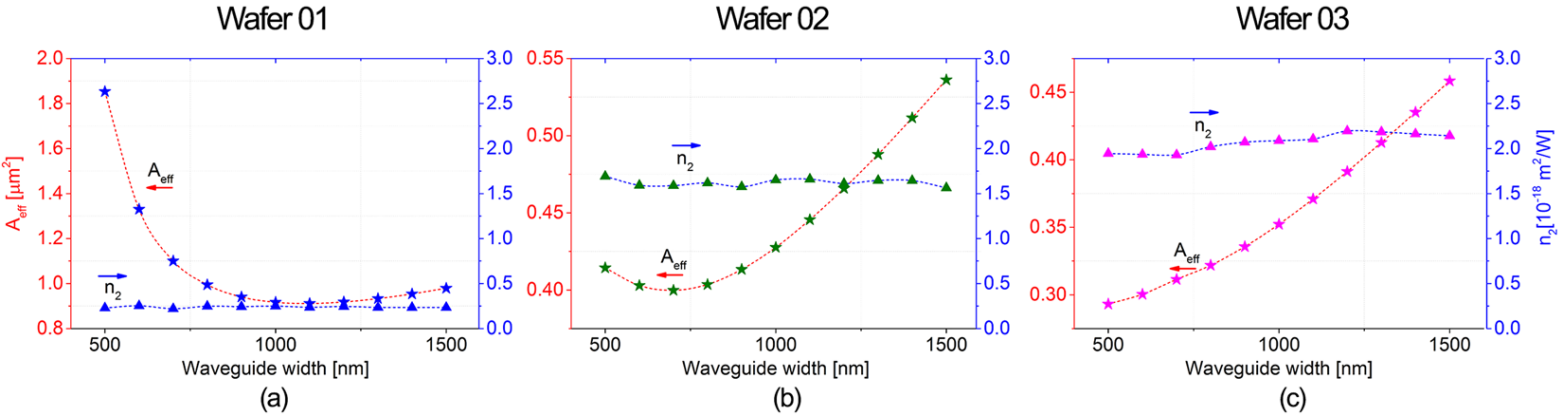
Calculated effective area and calculated n_2_ values for each waveguide width for each Si_x_N_y_ composition.

Blue dots represent Re{*γ*} coefficients obtained from the waveguides fabricated on Wafer 01, thus showing results for the standard, stoichiometric Si_3_N_4_. Green and purple dots represent data from Wafer 02 and Wafer 03 compositions, respectively. Re{γ} is influenced by the optical mode confinement achieved in each waveguide, which in turn depends on the waveguide dimensions. We calculated the effective area (*A*_*eff*_) for each waveguide configuration using the following equation^44,51^:

9 $${A}_{eff}=\frac{{a}_{NL}(\int {\int }_{-\infty }^{\infty }{S}_{z}dxdy)}{{\iint }_{NL}{S}_{z}dxdy}$$

where ${S}_{z}=(\overrightarrow{E}\times \overrightarrow{H})\cdot \hat{z}$ is the time averaged *z* component of the Poynting vector, *z* is the unit vector along the waveguide axis, *a*_*NL*_ is the core cross section area and *NL* denotes the integration over the nonlinear region. A Finite Difference Method (FDM) solver was used to calculate the electrical and magnetic field distributions. Using the following equation:

10 $${n}_{2}=\frac{\lambda }{2\pi }{\rm{Re}}\{\gamma \}{A}_{eff}$$

and taking into account the calculated *A*_*eff*_, the nonlinear refractive index of each wafer configuration can be extracted from the experimental data. Results are presented in Fig. 7 and the average calculated *n*_*2*_ values, for each Si_x_N_y_ configuration, are shown in Table 2 along with the value obtained by testing crystalline silicon waveguides. The calculated *A*_*eff*_ values (star symbols connected by red lines) show that the optical confinement is maximized at W = 1100 nm, W = 700 nm and W = 500 nm for Wafer 01, Wafer 02 and Wafer 03, respectively. These trends are reflected in the experimental data (Fig. 6) for which the Re{γ} values are maximized when *A*_*eff*_ values are at their lowest points.

**Table 2:** Extracted n_2_ values for 220-nm SOI, Wafer 01, Wafer 02 and Wafer 03 configurations.

Wafer ID	n_2_ [m^2^/W]
Silicon WG–calibration	(2.21 ± 0.1) × 10^−18^
Wafer 01	(2.3 ± 0.3) × 10^−19^
Wafer 02	(1.61 ± 0.2) × 10^−18^
Wafer 03	(2 ± 0.15) × 10^−18^

The value of *n*_*2*_ shown for Wafer 01 is in good agreement with data already reported in the literature for silicon nitride waveguides^30^. Wafers 02 and 03 show enhanced nonlinear refractive index (by an order of magnitude with respect to Wafer 01), confirming that the large enhancement observed in terms of Re{γ} (Fig. 6) is mainly due to the nonlinear properties of the material. Therefore, by properly tuning the silicon content in the Si_x_N_y_ layers the Re{*γ*} coefficient can be made to approach values close to 30 (Wm)^−1^. Finally, It is worth noting that the *n*_*2*_ value obtained for the crystalline silicon (calibration waveguides) is in good agreement with data reported by Lin *et al*.^12^.

**Figure 8:**
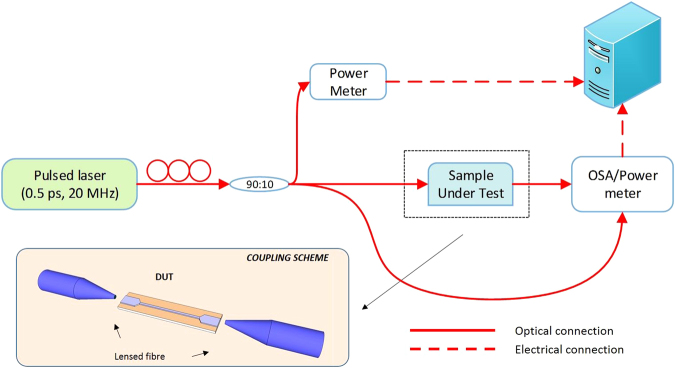
TPA experimental scheme.

The imaginary part of the nonlinear coefficient *γ(ω)* can be assessed by means of pulsed laser experiments, by following the procedure detailed in ref. 14. The experimental set-up is shown in Fig. 8; a fibre mode-locked laser centred at 1550 nm (model Calmar Mendocino Laser), with pulse duration of 0.5 ps, and repetition rate of 20 MHz was used as the signal source. In order not to impose any spectral shaping on the input pulses, a butt-coupling scheme was employed (see Fig. 8); a pair of lensed fibres (MDF = 3 μm) was used to couple light in and out of the waveguides under test. Coupling losses of 11 ± 0.5 dB/facet were estimated by means of low power CW transmission experiments. Polarization was set by means of fibre polarization controllers, placed at the output of the fibre laser.

By measuring the average power at the output of the sample under test as a function of the input peak power, the TPA-coefficients of the various waveguides were assessed. By assuming a hyperbolic-secant pulse temporal profile (which is the nominal pulse shape of our source), the average output power can be related to the input peak power through the following equation^14^:

11 $$\overline{P(L)}=\frac{\mathrm{ln}(\sqrt{\delta }+\sqrt{\delta +1})}{\sqrt{\delta (\delta +1)}}\overline{P(0)}{e}^{-\alpha L}$$

where $\overline{P(L)}$and $\overline{P(0)}$ are the average output power and the average input power, respectively and

12 $$\delta =\frac{{\alpha }_{TPA}}{{A}_{eff}}{L}_{eff}P{(0)}_{peak}$$

TPA-coefficients (thus Im{*γ*}) were assessed by using equations (11) and (12). In order to confirm that reasonable values for the TPA coefficients were obtained with our experimental procedure, we also carried out measurements on standard Si-waveguides (calibration set) and we obtained a value of (0.8 ± 0.06) cm/GW which is in good agreement with values reported in the literature^10^.

**Figure 9:**
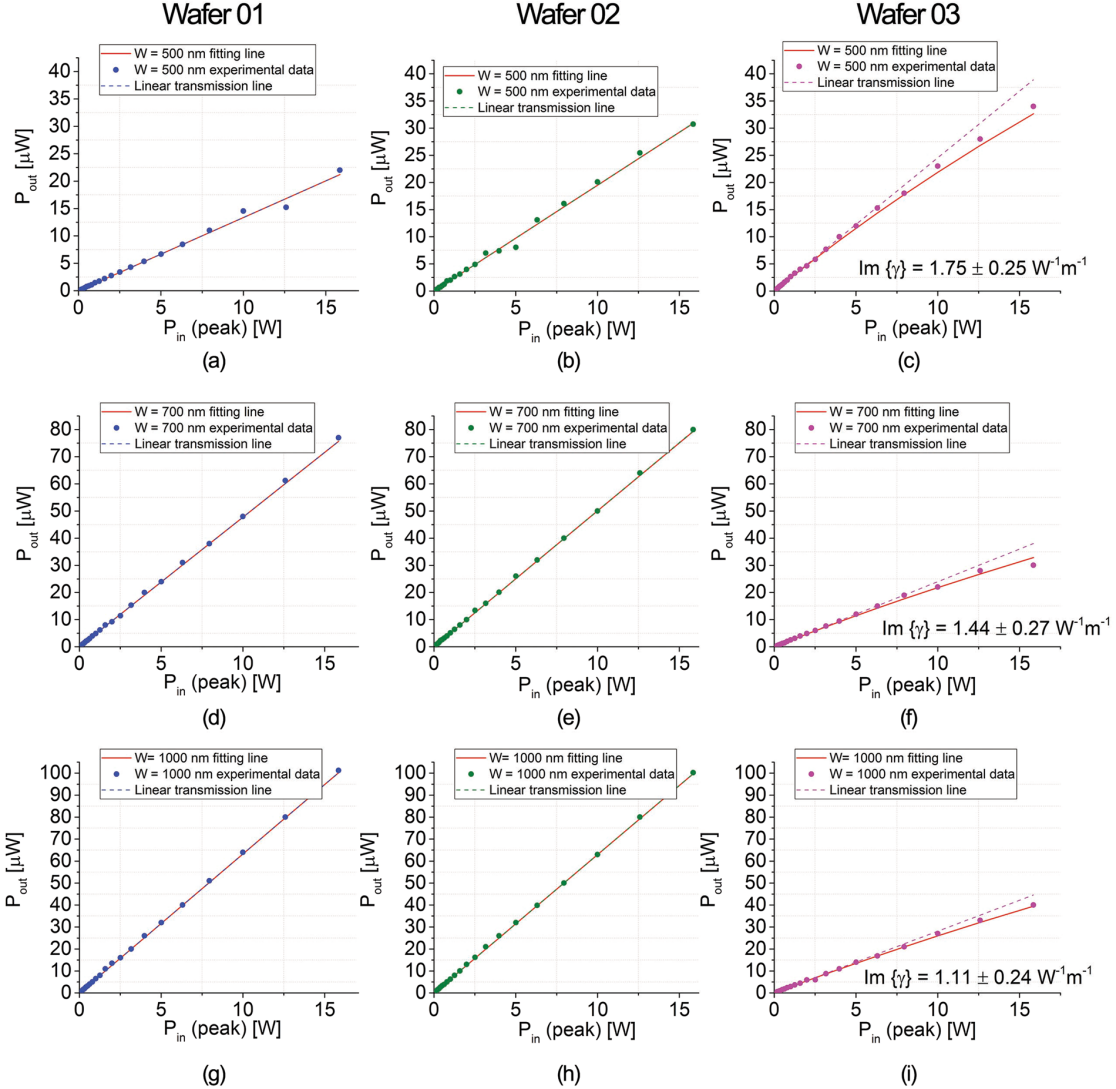
Pulsed transmission graph for Wafer 01 ((**a**) W = 500 nm, (**d**) W = 700 nm and (**g**) W = 1000 nm), Wafer 02 ((**b**) W = 500 nm, (**e**) W = 700 nm and (**h**) W = 1000 nm) and Wafer 03 ((**c**) W = 500 nm, (**f**) W = 700 nm and (**i**) W = 1000 nm).

Fig. 9 shows transmission experiment results for three selected waveguide-width configurations (W = 500 nm, 700 and 1000 nm), for the three different Si_x_N_y_ layer compositions. No TPA power saturation can be observed in waveguides fabricated on Wafer 01, suggesting negligible *α*_*TPA*_. Wafer 02 waveguides also do not exhibit any TPA (second column), while relatively small TPA-induced losses are seen in the waveguides fabricated on Wafer 03, suggesting that the TPA threshold has been exceeded and that a further increase in the Si percentage in the material might cause additional nonlinear losses.

By combining results obtained for each waveguide-width configuration, the *α*_*TPA*_ coefficient of each Si_x_N_y_ composition was determined and these results are shown in Table 3.

**Table 3:** TPA coefficients measured at 1550 nm.

Wafer ID	α_TPA_ [cm/GW] at λ = 1550 nm
Silicon WG	(0.8 ± 0.06)
Wafer 01	Negligible
Wafer 02	Negligible
Wafer 03	(0.1 ± 0.01)

## Discussion

The nonlinear phase shift caused by the interaction between a light beam and a third-order nonlinear device (which determines the efficiency of the device as a nonlinear medium) can be expressed as follows^50^:

13 $${\rm{\Delta }}\phi =\frac{1}{2}\frac{{\rm{Re}}\{\gamma \}}{{\rm{Im}}\{\gamma \}}\,\mathrm{ln}(1+2{\rm{Im}}\{\gamma \}{L}_{eff}{P}_{0})$$

where *P*_0_ represents the peak optical power at the input of the waveguide.

**Figure 10:**
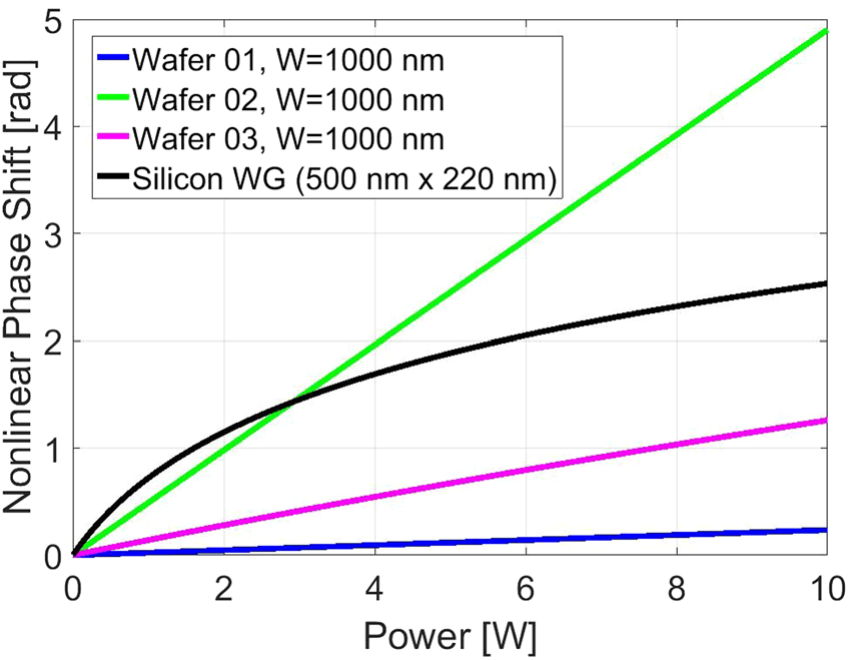
Calculated nonlinear phase shift for a L = 0.1 m waveguide.

A phase shift of π is typically required to implement optical processing functionalities such as optical modulation, wavelength and format conversion and optical regeneration^52^. In order to evaluate the overall performance of our Si_x_N_y_ nonlinear waveguides we have numerically calculated equation (13) for specific waveguide dimensions (width = 1000 nm, height = 300 nm *L* = 0.1 m) for the three Si_x_N_y_ different material compositions studied here. The results are shown in Fig. 10 and are contrasted against our internally fabricated Si waveguide of cross-sectional dimensions 500 nm × 220 nm (black line). As expected, even at a relatively low pump power levels (~1 W), TPA greatly reduces the nonlinear efficiency of the Si waveguide, showing a maximum saturated nonlinear phase shift of ~π/2. The blue line shows results achievable using a waveguide grown on Wafer 01 (stoichiometric silicon nitride Si_3_N_4_); thanks to the absence of TPA, no saturation effect is observed on the achievable phase shift, even at high power levels (>10 W). On the other hand, the relatively low Kerr coefficient, strongly limits the achievable amount of nonlinear phase shift in these waveguides, making this configuration not suitable for practical nonlinear applications. By greatly increasing the silicon content in the Si_x_N_y_ composition, the Kerr response can be enhanced as shown in Fig. 10. Wafer 03 is the richest in silicon content and the achievable nonlinear phase shift provided by waveguides grown on this material is represented by the magenta line. No noticeable TPA effects can be observed within the plotted range of powers, however the additional linear loss (Fig. 4, right-hand panel) introduced by the excess silicon limits *L*_*eff*_, thus reducing the overall nonlinear efficiency; a maximum, non-saturated, nonlinear phase shift of π/2 can be achieved in 0.1-m-long waveguides of this configuration. The green line shows results obtained for the Wafer 02 waveguide. Thanks to the absence of TPA, no phase shift saturation effect is observed; moreover, due to the enhanced Kerr response (see Fig. 10) and low linear loss (see Fig. 4(b)), the achievable nonlinear phase shift at these power levels, exceeds π, making this material suitable for the realization of practical telecom applications.

It is also worth noting that in the presence of strong TPA (i.e. in standard silicon waveguides, see black curve in Fig. 10), Free Carrier Absorption (FCA) also becomes significant, further reducing the nonlinear efficiency of these devices, making them unsuitable for operation at high power levels. The developed Si_x_N_y_ material composition does not suffer from any additional losses caused by FCA, and is therefore well suited both for high- and low-power applications.

Table 4 compares the properties of different platforms for integrated nonlinear optical devices that can be found in the literature to those of typical Si waveguides and the Wafer 02 waveguides of this work. Hydex® shows a record low loss of only 0.06 dB/cm and a remarkable nonlinear Kerr coefficient and no TPA at telecom wavelengths; the fabrication of this doped glass only requires low temperature process making it BEOL-CMOS compatible^18^. However, relatively long waveguides (in the orders of meters) are required to achieve a π-phase shift thus increasing the total device footprint. Silicon-based nonlinear devices have been widely studied during the last 10 years. As already discussed previously, silicon exhibits a very large Kerr coefficient that makes it attractive for the implementation of nonlinear devices; unfortunately, this is also typically accompanied by a relatively high nonlinear loss (TPA and FCA) that can prevent the operation of such waveguides in the high power regime (>500 mW). Amorphous silicon has also been intensively investigated by researchers, showing reduced TPA compared to c-Si. The main issues regarding this material seem to be the increased linear loss value (due to presence of silicon dangling bonds^14,15,48^ ,mitigated by hydrogen passivation) and material instability and degradation reported after high intensity light exposure^53^. A number of Si-based devices are reported in Table 4, showing that Re{γ} and Im{γ} can be significantly modified by a careful choice of fabrication conditions and waveguide geometries. Silicon germanium has also emerged in the last years as possible alternative to silicon^28,29^, however TPA and FCA are increased at telecom wavelength due to the presence of germanium, making this material a better choice for longer wavelengths. AlGaAs and Ta_2_O_5_ are also reported in Table 4, showing remarkable performance. Although these materials show very good nonlinear performance at telecoms wavelengths and are fully CMOS-compatible, they usually need high temperature annealing steps to reduce their linear loss, making them unsuitable for BEOL fabrications. Finally we report a number of devices based on silicon nitride. Record low loss have been demonstrated by Bauters *et al*.^33^ (high aspect ratio waveguides) and several nonlinear waveguides have been shown in the literature^19,34,37^. However they are usually fabricated using LPCVD that requires high temperature steps. This reason has pushed research towards the development of PECVD Si_x_N_y_ devices that may offer comparable performance without the need for high temperature treatment. A very large Re{*γ*} has been demonstrated in ref. 36, however this was also accompanied by a high propagation loss (10 dB/cm) that limits the maximum effective waveguide length to a mm scale. Low losses were demonstrated in ref. 38, however the nonlinearity of these waveguides was rather low, thus limiting the achievable phase shift when cm-scale waveguides are used. Furthermore, the material used in ref. 38 required high temperature processing, which makes it not suitable for BEOL-CMOS-compatible processes. In contrast, the nonlinear coefficient Re{*γ*} of the waveguides presented herein has increased by about 3 times with respect to^38^, while at the same time, low loss and BEOL-CMOS-compatibility has been ensured, providing a novel platform, suitable for nonlinear optical processing experimentation.

**Table 4:** Key parameters for different nonlinear waveguide technologies.

	Re{γ}[W^−1^m^−1^]	Im{γ}[W^−1^m^−1^]	α [dB/cm]	1/α [cm]	CMOS-BEOL compatibility
Hydex ®^19,20^	0.23	Negligible	0.06	138.15	Yes
Si-based^4,9–12,14,15,17,54–62^	60–1100	20–90	1–6	0.5–5	Yes
Silicon Germanium^28^	25	3.3	1.4	3.10	Yes
AlGaAs^22,23,25,63,64^	10–600	Negligible	1.2–6	3.61–0.72	No
Ta_2_O_5_^26,27^	0.1–5	Negligible	0.15–1.5	2.8–28	No
Low loss silicon nitride^19,31,33,34,65^	0.1–1.5	Negligible	0.02–1	4–200	No
Si-WG (500 nm × 220 nm)–This work, used as calibration medium	70	35	3	1.44	Yes
Si-rich Si_x_N_y_–T Wang *et al.* ^36^	550	Negligible	10	0.43	Yes
Si-rich Si_x_N_y_ L. Xing *et al.* ^38^	5.7	Negligible	1.35	3.22	No
**This Work (Wafer 02 Configuration, W** = **1000 nm)**	**16**	**Negligible**	**1.5**	**2.9**	**Yes**

## Conclusion

We presented a complete characterization of the linear and nonlinear properties of BEOL-CMOS-compatible Si_x_N_y_ waveguides. We showed that the material can be engineered such that it can exhibit properties that are well-suited for operation at either low of high power levels. The fabrication of Si-rich silicon nitride waveguides based on the PECVD technique at relatively low temperatures (350 °C), with a propagation loss of approximately 1.5 dB/cm was reported. We also demonstrated that the nonlinear Kerr response can be engineered and increased by an order of magnitude increase, in terms of *n*_*2*_, by optimizing the fabrication parameters. TPA can be kept negligible at 1550 nm, enabling high power operation, which is normally prevented in typical Si waveguides. A nonlinear phase shift of π can be obtained by using the developed Si_x_N_y_ material composition, making it suitable for the realization of telecom devices for advanced nonlinear applications in next generation optical networks.

## Methods

### Wafer fabrication

The Si_x_N_y_ layers were fabricated starting from a 150 mm diameter SiO_2_-on-Si substrate wafer. Nitride layers were deposited using a PECVD tool (model OIPT SYS 100), by employing the parameters specified in Table 5. The chamber pressure and RF power where set to 980 mTorr and 60 W, respectively. After deposition, waveguides where written using a waveguide layout designed to provide different test structures, including straight waveguides, spiral waveguides and fibre-to-chip coupling devices. Both grating couplers and butt-couplers were designed on the mask in order to provide access to the waveguides using either of the two methods. The mask layout was transferred to the wafers using E-beam patterning (positive electron beam resist ZEP 520A was used). After the development of photoresist, ICP etching was performed in order to define the waveguide patterns on the light guiding layer. The remaining resist was removed by ashing in oxygen plasma. The protective, 1 μm-thick, SiO_2_ cladding layer was finally deposited by PECVD using a standard recipe for the deposition of stoichiometric SiO_2_. Finally, the samples were cleaved to provide access to the butt-coupling devices. Standard Si- waveguides were also used as a reference. The standard silicon waveguide fabrication started from a 150mm SOI commercial wafer (220 nm top Si layer thickness). Waveguides were patterned and fabricated using the same processing steps as described above.

**Table 5:** PECVD deposition parameters for the three different wafer composition employed in this work.

Wafer ID	SiH_4_ (sccm)	NH_3_ (sccm)	N_2_ (sccm)	Platen Temperature (°C)
Wafer 01	1.8	0	980	350
Wafer 02	3.6	0	980	350
Wafer 03	7.2	0	980	350

### Error evaluation procedure

In order to provide statistically significant results, each measurement presented in the paper was performed on a minimum number of 100 nominally identical waveguides. Error bars have been included in the graphs presented in the paper, which have taken into account both systematic errors and curve fitting standard deviations. The highest and lowest measured values were excluded from the calculation of each value showed in the manuscript. The detailed procedures adopted to evaluate the mean value of each measurement and its associated error are described below.

#### Propagation loss measurements

The propagation losses were evaluated over the three Si_x_N_y_ wafer compositions, for different waveguide widths (ranging from 500 nm to 1500 nm, with a step of 100 nm). Each propagation loss value was estimated using cut back measurements, performed on a set of 11 waveguides of different lengths (0.1 cm to 1.1 cm, with a step of 0.1 cm). The result of each cut-back measurement provided as output a fitted value (the propagation loss of a single waveguide width) and its associated standard deviation (α_i_ ± σ_i_). One hundred different cut back measurements were performed on one hundred, nominally identical, waveguide sets, thus providing a set of one hundred measurement results as follows: [α_1_ ± σ_1_, α_2_ ± σ_2_, α_3_ ± σ_3_,_ ………_ α_100_ ± σ_100_]. The final, averaged, value was calculated as follows:

14 $$\overline{\alpha }\pm \overline{\sigma }=\mathop{\underbrace{\frac{{\alpha }_{1}+{\alpha }_{2}+{\alpha }_{3}+\mathrm{....}+{\alpha }_{100}}{100}}}\limits_{\overline{\alpha }}\pm \mathop{\underbrace{\sqrt{{\sigma }_{1}^{2}+{\sigma }_{2}^{2}+{\sigma }_{3}^{2}+\mathrm{....}+{\sigma }_{100}^{2}}}}\limits_{\overline{\sigma }}$$

#### Re{*γ*} measurements

Kerr coefficients were evaluated for each waveguide width (500 nm to 1500 nm) across the three different Si_x_N_y_ material wafers. According to equation (8), Re{*γ*} can be extracted by measuring the CW-FWM efficiency, at different pump power levels. For each measurement we used a minimum number of 40 different pump power levels to provide a power versus FWM-efficiency curve that can be fitted by a second order polynomial curve, which provides as an output the value of Re{*γ*} and its standard deviation *σ*. For each waveguide-width configuration, the measurement was repeated on one hundred nominally identical waveguides (*L* = 1 cm), providing a set of values: [Re{*γ*}_1_ ± *σ*_1_, Re{*γ*}_2_ ± *σ*_2_, Re{*γ*}_3_ ± *σ*_3_, …., Re{*γ*}_100_ ± *σ*_100_]. The averaged values, for each single waveguide, along with their standard deviation values were calculated as follows:

15 $$\begin{array}{lll}\overline{{\rm{Re}}\{\gamma \}}\pm \overline{\sigma } & = & \mathop{\underbrace{\frac{{\rm{Re}}{\{\gamma \}}_{1}+\mathrm{Re}{\{\gamma \}}_{2}+{\rm{Re}}{\{\gamma \}}_{3}+\mathrm{....}+{\rm{Re}}{\{\gamma \}}_{100}}{100}}}\limits_{\overline{{\rm{Re}}\{\gamma \}}}\\  &  & \pm \mathop{\underbrace{\sqrt{{{\sigma }_{1}}^{2}+{{\sigma }_{2}}^{2}+{{\sigma }_{3}}^{2}+\mathrm{....}+{{\sigma }_{100}}^{2}}}}\limits_{\overline{\sigma }}\end{array}$$

#### Im{*γ*} measurements

The Im{*γ*} coefficient was assessed by employing an optical pulse transmission set-up, for each waveguide (for each different width), across the three Si_x_N_y_ wafers. For a single measurement we used a minimum of 40 input power levels. Each measurement session provided a single value of Im{*γ*} and its standard deviation *σ*. We repeated the same measurement session on one hundred nominally identical waveguides (*L* = 1 cm), producing a set of values: [Im{*γ*}_1_ ± *σ*_1_, Im{*γ*}_2_ ± *σ*_2_, Im{*γ*}_3_ ± *σ*_3_, …., Im{*γ*}_100_ ± *σ*_100_]. The averaged Im{*γ*} values, for each single waveguide, along their standard deviation values were calculated as follows:

16 $$\begin{array}{lll}\overline{{\rm{Im}}\{\gamma \}}\pm \overline{\sigma } & = & \mathop{\underbrace{\frac{{\rm{Im}}{\{\gamma \}}_{1}+{\rm{Im}}{\{\gamma \}}_{2}+{\rm{Im}}{\{\gamma \}}_{3}+\mathrm{....}+{\rm{Im}}{\{\gamma \}}_{100}}{100}}}\limits_{\overline{{\rm{Im}}\{\gamma \}}}\\  &  & \pm \mathop{\underbrace{\sqrt{{{\sigma }_{1}}^{2}+{{\sigma }_{2}}^{2}+{{\sigma }_{3}}^{2}+\mathrm{....}+{{\sigma }_{100}}^{2}}}}\limits_{\overline{\sigma }}\end{array}$$
